# EHMT2 inhibitor BIX-01294 induces apoptosis through PMAIP1-USP9X-MCL1 axis in human bladder cancer cells

**DOI:** 10.1186/s12935-014-0149-x

**Published:** 2015-02-04

**Authors:** Jing Cui, Wendong Sun, Xuexi Hao, Minli Wei, Xiaonan Su, Yajing Zhang, Ling Su, Xiangguo Liu

**Affiliations:** Shandong University School of Life Sciences, Room 103, South Building, 27 Shanda South Road, Jinan, 250100 China; The Second Hospital, Shandong University, Jinan, China

**Keywords:** Apoptosis, PMAIP1, MCL1, USP9X, DDIT3, ER stress, EHMT2, BIX-01294

## Abstract

BIX-01294, an euchromatic histone-lysine N-methyltransferase 2 (EHMT2) inhibitor, has been reported to induce apoptosis in human neuroblastoma cells and inhibit the proliferation of bladder cancer cells. However, the definite mechanism of the apoptosis mediated by BIX-01294 in bladder cancer cells remains unclear. In the present study, we found that BIX-01294 induced caspase-dependent apoptosis in human bladder cancer cells. Moreover, our data show BIX-01294 stimulates endoplasmic reticulum stress (ER stress) and up-regulated expression of PMAIP1 through DDIT3 up-regulation. Furthermore, down-regulation of the deubiquitinase USP9X by BIX-01294 results in downstream reduction of MCL1 expression, leading to apoptosis eventually. Thus, our findings demonstrate PMAIP1-USP9X-MCL1 axis may contribute to BIX-01294-induced apoptosis in human bladder cancer cells.

## Introduction

EHMT2, also known as G9a, is an important enzyme for histone H3 dimethylation at lysine-9 [1,2]. EHMT2 is overexpressed in breast, prostate, colon, bladder, ovarian, melanoma, lung, and liver cancers [3-5]. Recent research shows that enhanced expression of EHMT2 is involved in the proliferation of cancer cells [6]. BIX-01294, a diazepin-quinazolinamine derivative, is a specific inhibitor of EHMT2 by reducing the H3K9Me2 level [7]. Furthermore, BIX-01294 is the first small compound that can replace Oct4 to induce neural stem cells to be reprogrammed into iPSCs [8]. Moreover, BIX-01294 can induce apoptosis in human neuroblastoma cells by raising CASP8 and CASP3 activity [9]. BIX-01294 also can decrease the proliferating activity of bladder cancer cells [6]. It was reported that knockdown of *EHMT2* by siRNA [3,4,10,11] or treatment with BIX-01294 [10,12,13] inhibited growth of bladder, lung, prostate, and breast cancer cells.

A variety of detrimental stimuli, such as ultraviolet light, hypoxia, oxidative stress, can affect the function of ER (endoplasmic reticulum), leading to accumulation of unfolded or misfolded protein in ER and activation of ER stress response [14-18]. A large number of unfolded or misfolded proteins can bind to HSPA5, and then activate three pathways through PERK, ERN1 and ATF6. The long-term, severe ER stress will ultimately induce cell apoptosis [19]. DDIT3 is a transcriptional factor and the downstream member of ER stress pathways [20,21]. Recent studies have shown that numerous drugs, such as lonafarnib, MG132, CDDO-Me and pemetrexed up-regulate DDIT3 expression via p-EIF2S1-ATF4 axis and accordingly enhance apoptosis through induction of DR5 or Bim etc. [22-24].

Mitochondrion is at the center of intrinsic apoptotic pathway. Under the regulation of BCL-2 family, Cyt c, a component of mitochondrial electron transport chain, can be released into cytoplasm through a hole related to the oligomerization of BAX/BAK and VDAC (voltage-dependent anion channel). The Cyt c in cytoplasm can combine with APAF1 and induce cell apoptosis [25]. MCL1 drives its pro-survival function by combining with BAX/BAK and inhibiting the oligomerization. PMAIP1 belongs to the pro-apoptotic BH3-only family and has been known to suppress pro-survival MCL1 and A1 through binding to them [26]. Several BH3-only family proteins, including PMAIP1, can be activated by DDIT3 [27]. Notably, PMAIP1 can control the stability of MCL1 through regulating its polyubiquitination which E3 ligase HUWE1 or FBW7 are involved in [28]. Ubiquitin specific peptidase 9x (USP9X), a deubiquitinase belonging to USP family, can stabilize MCL1 through removing the polyubiquitin chains linked by Lys-48 which is the essential sign in proteasomal degradation process, and it is overexpressed in several kinds of cancer cells [29]. Moreover, over-expression of PMAIP1 may trigger a decrease in the USP9X/MCL1 interaction [28]. Recent studies also show that high level of PMAIP1 likely reduces the availability of USP9X to MCL1, thereby promoting the ubiquitination and degradation of MCL1, leading to the apoptosis of neoplastic cells [30].

However, the specific mechanism of apoptosis mediated by BIX-01294 in bladder cancer cells remains unknown. The goal of our research was to investigate which pathway BIX-01294 follows to induce apoptosis in human bladder cancer cells and which proteins play important roles in this process. In our research, BIX-01294 was capable of leading to ER stress. Furthermore, DDIT3, as a downstream transcriptional factor in ER stress, promotes the expression of PMAIP1 which can significantly lower MCL1 levels. Moreover, BIX-01294 also reduces USP9X and then affects the stability of MCL1. According to our present study, we demonstrate that BIX-01294 causes apoptosis in bladder cancer cells through ER stress pathway, resulting in PMAIP1 up-regulation and MCL1 down-regulation.

## Materials and methods

### Reagents

The powder of BIX-01294 (B9311) trihydrochloride and EHMT2 (G6919) antibody were purchased from Sigma-Aldrich (City of Saint Louis, Missouri, US). CASP3 (IMG-145) antibody was purchased from Imgenex. USP9X (5751), DDIT3 (2895), CASP8 (9746), CASP9 (9502), PARP1 (9542), HSPA5 (3183) and ERN1 (3294) antibodies were purchased from Cell Signaling Technology (Boston, Massachusetts, US). ATF4 (SC-200) and MCL1 (SC-12756) antibodies were obtained from Santa Cruz Biotechnology (Santa Cruz, California, US). PMAIP1 (OP180) antibody was purchased from Calbiochem (San Diego, California, US).

### Cell lines and cell culture

The human bladder cell lines T24 and 5637 were obtained from the Cell Bank of Shanghai Institute of Biochemistry and Cell Biology, Chinese Academy of Sciences (Shanghai, China). T24 and 5637 were grown in monolayer culture at 37°C in a humidified atmosphere consisting of 5% CO_2_ and 95% air. The T24 cell line was cultured in McCoy's 5A Modified Media (Sigma-Aldrich) supplemented with 5% (v/v) FBS (SAFC®Global) and the 5637 cell line was cultured in RPMI 1640 (Sigma-Aldrich) supplemented with 5% (v/v) FBS.

### Western blot analysis

Preparation of whole cell protein lysates and the procedures for the Western blot analysis were already described [31].

### siRNA transfections

All the siRNAs were synthesized by GenePharma. The small interfering RNA (siRNA) duplexes for control, *DDIT3* siRNA duplexes target the sequence 5'-GCCTGGTATGAG GACCTGC-3' [32]. *EHMT2* siRNA duplexes target the sequence 5′-GCATTTCCGCATGAGTGAT-3′. *PMAIP1* siRNA duplexes target the sequence 5′-GGAAGUCGAGUGUGCUACU-3′, *USP9X* siRNA duplexes target the sequence 5′-CAATCAAGTTCAATGATTA-3′, The transfection of siRNA was conducted with X-tremeGENE siRNA Transfection Reagent (Roche) following the instructions.

### Cell survival assay

On the first day, cells were seeded in 96 well plates. Twenty-four hours later, BIX-01294 was added at the indicated concentrations in each well. After treatment by BIX-01294 for another 24 h, we used 10% TCA to immobilize the cells and then evaluated the living cell numbers using the sulforhodamine B assay as previously described [33].

### Apoptosis assays

Apoptosis was detected by the Annexin V-FITC Apoptosis Detection Kit (BIOBOX) following the manufacturer’s protocol. Activation of the caspase family was evaluated by western blot.

### Construction of the plasmid

*MCL1* gene was amplified from A549 cells cDNA with PCR technique using the primers described as below: 5′-CGGATCCGCCGCCACCATGTGTTTGGCCTCAAAAGAAACG-3′ (sense); 5′-CGGGCCCCTATCTTATTAGATATGCCAAAC-3′ (antisense). This resultant construct was named pcDNA3.1-MCL1 after *MCL1* gene was cloned into pcDNA3.1 (+) vector by using *Bam* HI and *Apa* I restriction sites [30].

### Plasmid transient transfections

On the first day, T24 and 5637 cells were seeded into 6-well plates. On the second day, cells were transfected with pcDNA3.1 and pcDNA3.1-MCL1 using X-tremeGENE HP DNA Transfection Reagent (Roche). On the third day, experimental groups were treated by 10 μM BIX-01294 for 24 hours. On the fourth day, the cells were harvested for western blot.

## Results

### BIX-01294 induces caspase-dependent apoptosis in human bladder cancer cell lines

**Figure 1:**
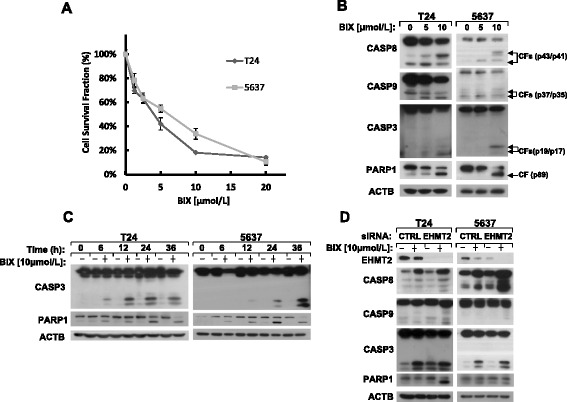
**BIX-01294 induces apoptosis in human bladder cancer cells. (A)** The indicated cell lines were seeded in 96-well plates on the first day and treated with 1.25, 2.5, 5, 10 and 20 μmol/l of BIX-01294 on the second day. After treatment for 24 hours, live cell number was estimated using SRB assay for calculation of cell survival. Points: mean of four replicate determinations; bars: S.D. **(B)** T24 and 5637 cells were treated with indicated concentrations of BIX-01294 for 24 hours and harvested for Western blot analysis. Levels of protein expression were analyzed by using antibodies against CASP8, CASP9, CASP3, PARP1 and ACTB. **(C)** T24 and 5637 cells were treated with 10 μmol/l of BIX-01294 for various lengths of time. Both attached and suspended cells were harvested for Western blot analysis. Levels of protein expression were analyzed by using antibodies against CASP8, CASP3, PARP1 and ACTB. CF: cleaved form. **(D)** Forty-eight hours after control siRNA and *EHMT2* siRNA transfection, cells were treated with 10 μmol/l of BIX-01294. Whole-cell protein lysates were harvested for Western blot analysis. Levels of protein expression were analyzed by using antibodies against EHMT2, CASP8, CASP9, CASP3, PARP1 and ACTB.

We examined BIX-01294’s biological effect in cell lines T24 and 5637 to figure out whether this agent induced caspase-dependent apoptosis in human bladder cancer cells. Cell survival rate was determined by SRB assays (Figure 1A) after the cells were treated with 1.25, 2.5, 5, 10 or 20 μmol/l of BIX-01294 for 24 hours. The data show that BIX-01294 inhibited the cell proliferation in a concentration-dependent manner in T24 and 5637 cells. Western blot analysis demonstrates that BIX-01294 (0, 5, 10 μmol/l) significantly activated CASP8, CASP9, CASP3 and cleaved the substrate of CASP3, PARP1 in bladder cancer cells treated for 24 hours (Figure 1B). At the same concentration, it is observed that the cleavage of caspases and PARP1 increased gradually over time (Figure 1C). To characterize whether the apoptosis induced by BIX-01294 is related to EHMT2, we treated cells with BIX-01294 after the cells were transfected with *EHMT2* siRNA. As shown in Figure 1D, the cleaved caspases and PARP1 increased apparently upon the inhibition of EHMT2 expression. In summary, BIX-01294, as a specific inhibitor of EHMT2, induces caspase-dependent apoptosis in a dose- and time-dependent manner in human bladder cancer cells.

### BIX-01294 induces up-regulation of PMAIP1 and down-regulation of MCL1 in human bladder cancer cells

**Figure 2:**
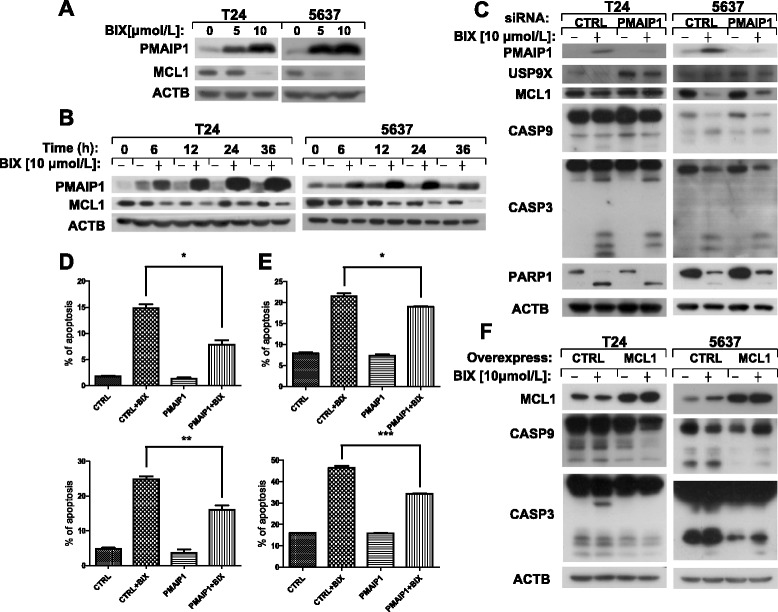
**BIX-01294 up-regulates the expression of PMAIP1 and down-regulates MCL1 in bladder cancer cells.** T24 and 5637 cells were treated with indicated concentrations of BIX-01294 for 24 hours or the cells were treated with 10 μmol/l of BIX-01294 for various lengths of time. Both attached and suspended cells were harvested for Western blot analysis **(A and B)**. We used control siRNA and *PMAIP1* siRNA to transfect T24 **(C and D)** and 5637 **(C and E)** for 48 hours and then added BIX-01294 (10 μmol/l) into culture medium and harvested the cells for western blot analysis by using antibodies against PMAIP1, MCL1, CASP9, CASP3, PARP1, DDIT3 and ACTB or for detection of apoptotic cells using Annexin V-FITC staining. In **D** and **E**, the early apoptosis detected by flow cytometry is the upper one and the total apoptosis is the lower one. MCL1, CASP9, CASP3 and ACTB were detected by western blot after T24 and 5637 cells transfected with pcDNA3.1 and pcDNA3.1-MCL1 **(F)**. Columns: mean of triplicate treatments; bars: ± SD. The statistical differences between the two treatments were analyzed by two-sided unpaired Student’s t-tests (*p < 0.05; **p < 0.01; *** p < 0.001).

PMAIP1, a member of pro-apoptotic BH3-only protein family, is located at the outer mitochondrial membrane and plays critical role in the apoptosis induced by many chemotherapeutic agents in cancer cells [34]. As a member of anti-apoptotic proteins in BCL-2 family, MCL1 is overexpressed in many kinds of human cancers. In our model, BIX-01294 increased the expression of PMAIP1 and decreased the level of MCL1 in a concentration- and time-dependent manner in human bladder cells (Figure 2A and 2B). Moreover, *PMAIP1* knockdown with siRNA dramatically eased the alteration of USP9X and rescued the changes of MCL1, CASP9, CASP3 and PARP1 (Figure 2C). The fraction of apoptotic cells in PMAIP1 knockdown cells was reduced compared with that in control siRNA knockdown cells after BIX-01294 exposure according to the results of flow cytometry analysis (Figure 2D and 2E). To determine the importance of MCL1 in the process of apoptosis induced by BIX-01294, we overexpressed *MCL1* gene in human bladder cancer cells and then treated with BIX-01294 for 24 hours. Subsequent western blot analysis showed that overexpression of MCL1 attenuated apoptosis induced by BIX-01294 in T24 and 5637 cells (Figure 2F). These data suggest that PMAIP1 and MCL1 play important role in apoptosis induced by BIX-01294 in bladder cancer cells.

### BIX-01294 reduces MCL1 expression through a USP9X-dependent mechanism

**Figure 3:**
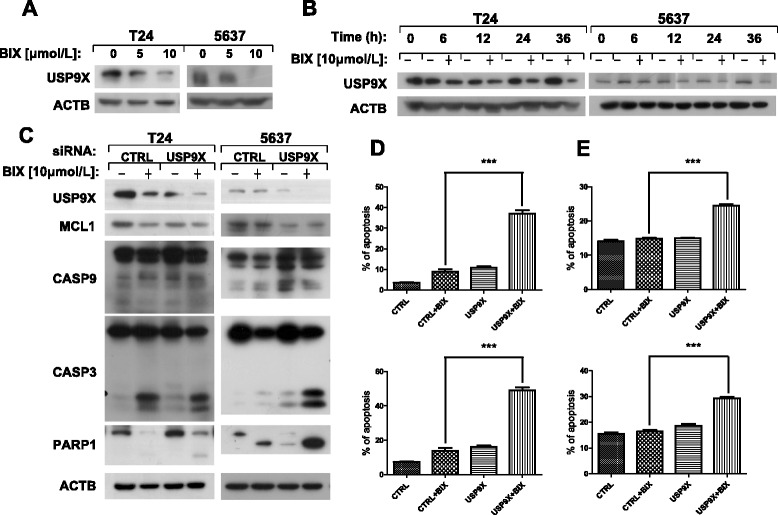
**BIX-01294 reduces USP9X level in the concentration- and time-dependent manner, and USP9X knockdown enhances MCL1 degradation and apoptosis.** T24 and 5637 cells were treated with indicated concentrations of BIX-01294 for 24 hours or the cells were treated with 10 μmol/l of BIX-01294 for various lengths of time. Both attached and suspended cells were harvested for Western blot analysis **(A and B)**. After control siRNA and *USP9X* siRNA transfection, cells were treated with 10 μmol/l of BIX-01294 for 24 hours. We used antibodies against USP9X, MCL1, CASP9, CASP3, PARP1 and ACTB for western blot analysis **(C)** or used Annexin V-FITC Apoptosis Detection Kit for detection of apoptotic cells in T24 **(D)** and 5637 **(E)**. In **D** and **E**, the early apoptosis detected by flow cytometry is the upper one and the total apoptosis is the lower one. Columns: mean of triplicate treatments; bars: ± SD. The statistical differences between the two treatments were analyzed by two-sided unpaired Student’s t-tests (*p < 0.05; **p < 0.01; *** p < 0.001).

Remarkably, BIX-01294 can enhance the amount of PMAIP1 and reduce MCL1 in bladder cancer cells (Figure 2A and 2B). These data lead us to think whether BIX-01294 can influence the action of USP9X [35]. We found that BIX-01294 reduced the level of USP9X in the dose- and time-dependent manner (Figure 3A and 3B), and MCL1 was degraded more in *USP9X* siRNA knockdown cells (Figure 3C) compared with the Control siRNA knockdown cells. In addition, BIX-01294 induced less apoptosis in Control siRNA knockdown bladder cancer cells than that in *USP9X* siRNA knockdown cells (Figure 3D and 3E). Taken together, BIX-01294 decreases the expression of USP9X and promotes the degradation of MCL1, which eventually leads to apoptosis in bladder cancer cells.

### DDIT3 is up-regulated by BIX-01294 and regulates the expression of PMAIP1

**Figure 4:**
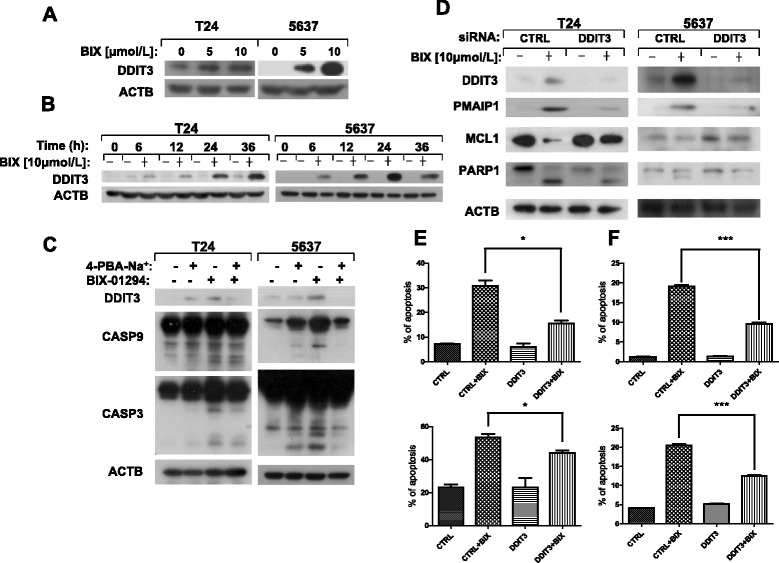
**BIX-01294 up-regulates the level of DDIT3, and DDIT3 knockdown suppresses the apoptosis induced by BIX-01294 in human bladder cancer cells.** T24 and 5637 cells were treated with indicated concentrations of BIX-01294 for 24 hours or the cells were treated with 10 μmol/l of BIX-01294 for various lengths of time. Both attached and suspended cells were harvested for Western blot analysis **(A and B)**. T24 and 5637 cells were pretreated with 4-PBA-Na^+^ (1 mM) for 30 min and then cotreated with BIX-01294 (10 μM) for another 24 h. Levels of protein expression were analyzed by western blot using antibodies against DDIT3, CASP9, CASP3 and ACTB **(C)**. After control siRNA and *DDIT3* siRNA transfection, cells were treated with 10 μmol/l of BIX-01294 for 24 hours. Whole-cell protein lysates were harvested for Western blot analysis by using antibodies against DDIT3, PARP1, PMAIP1, MCL1 and ACTB **(D)**. T24 **(E)** and 5637 **(F)** cells were transfected with control siRNA and *DDIT3* siRNA for 48 hours. After treatment with BIX-01294 (10 μmol/l) for 24 h, cells were stained with Annexin V-FITC/PI and detected by flow cytometry analysis. In **E** and **F**, the early apoptosis detected by flow cytometry is the upper one and the total apoptosis is the lower one. Columns: mean of triplicate treatments; bars: ± SD. The statistical differences between the two treatments were analyzed by two-sided unpaired Student’s t-tests (*p < 0.05; **p < 0.01; *** p < 0.001).

It is reported that as a transcriptional factor DDIT3 can induce apoptosis through surge of the BH3-only family members, including PMAIP1 [36]. Accordingly, we would like to figure out whether the expression of PMAIP1 induced by BIX-01294 is due to DDIT3. As we speculated, BIX-01294 was able to increase DDIT3 expression level (Figure 4A and 4B). In addition, we pretreated T24 and 5637 cells with ER stress antagonist 4-PBA (1 mM) for 30 minutes followed by incubation with BIX-01294 (10 μM) for another 24 hours and then measured the expression of DDIT3, CASP9, CASP3 and ACTB. The data showed that co-incubation with 4-PBA and BIX-01294 decreased the cleaved form of CASP9 and CASP3 (Figure 4C). In order to verify whether BIX-01294 triggers apoptosis through DDIT3, we used siRNA technique to silence DDIT3 expression and found that BIX-01294–induced cleavage of PARP1 was declined significantly (Figure 4D). Besides, we observed reduced expression of PMAIP1 and enhanced expression of MCL1 in DDIT3 knockdown cells after exposure to BIX-01294, suggesting that DDIT3 regulates PMAIP1 and MCL1. Consistently, BIX-01294 induced less apoptotic percentage in *DDIT3* siRNA knockdown T24 and 5637 cells than that in Control siRNA knockdown ones according to Annexin V-FITC staining and flow cytometry analysis (Figure 4E and 4F). To sum up, DDIT3, as a downstream member of ER stress pathway, enhances PMAIP1 expression and contributes to BIX-01294–induced apoptosis.

### BIX-01294 activates ER stress response

**Figure 5:**
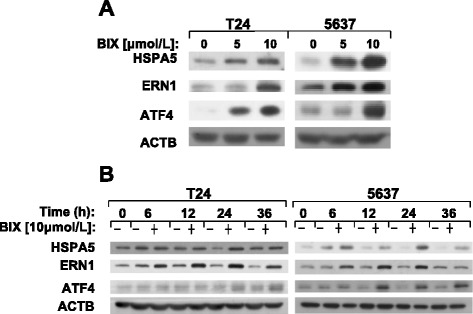
**BIX-01294 triggers ER stress response in human bladder cancer cells. (A)** T24 and 5637 cells were treated with indicated concentrations of BIX-01294 for 24 hours and harvested for Western blot analysis. Levels of protein expression were analyzed by using antibodies against HSPA5, ERN1, ATF4 and ACTB. **(B)** T24 and 5637 cells were treated with 10 μmol/l of BIX-01294 for various lengths of time. Both attached and suspended cells were harvested for Western blot analysis by using antibodies against HSPA5, ERN1, ATF4 and ACTB.

Since DDIT3 is a hallmark of ER stress, we wonder whether BIX-01294 induces apoptosis through ER stress. Moreover, DDIT3 expression strongly depends on ATF4 [37]. In our study, a number of proteins related to ER stress, like HSPA5, ERN1 and ATF4, were increased after treatment with BIX-01294 for 24 hours in T24 and 5637 cells (Figure 5A and 5B). In addition, we treated T24 and 5637 cancer cells with BIX-01294 for different lengths of time. As shown in Figure 5B, these proteins were up-regulated by a time-dependent manner after treated with BIX-01294. So far, we have figured out that BIX-01294 induces the expression of DDIT3 through activating ER stress, leading to up-regulation of PMAIP1.

## Discussion

To date, over-expression of EHMT2 may be a prognostic marker for patients with melanoma and esophageal cancer [38,39]. In addition, EHMT2 inhibitors can prevent the proliferation and self-renewal of AML via reducing HoxA9-dependent transcription and also can increase the intracellular ROS [7,40]. BIX-01294 has been demonstrated to induce autophagy in breast and colon cancers [7]. Earlier reports showed BIX-01294 led to apoptosis by activating CASP8 and CASP3 in human neuroblastoma cells and reduce the proliferation of human bladder cancer cells. In our study, with the increase of BIX-01294 concentration, the cell survival rate of bladder cancer cells T24 and 5637 reduced clearly. Moreover, by treating with BIX-01294, the expression level of some apoptosis-related proteins such as CASP8, CASP9, CASP3 and PARP1 were activated simultaneously. This means that BIX-01294 indeed induces the apoptosis in human bladder cancer cells. However, the mechanisms underlying how BIX-01294 triggers apoptosis in human bladder cancer cells remain unclear.

Cellular apoptosis can be induced by a long-term, severe ER stress. Some earlier studies have found that DDIT3, as a downstream component of ER stress, can be activated by ATF4. We also discovered that ER stress component, like HSPA5, ERN1, ATF4 and DDIT3, increased when the human bladder cancer cells were treated with BIX-01294. Moreover, the cleavage of PARP1 were suppressed in cells transfected with *DDIT3* siRNA. This implies the BIX-01294 induces caspase-dependent apoptosis through activating ER stress. Notably, the expression of PMAIP1 was reduced, while MCL1 was rescued when the cells were transfected with *DDIT3* siRNA. These results indicate that DDIT3 induces the expression of PMAIP1 which is required for BIX-01294-induced apoptosis.

In terms of mitochondrial apoptotic pathway, PMAIP1 belongs to the BH3-only pro-apoptotic protein family while MCL1 belongs to pro-survival BCL2 protein family. MCL1 can be suppressed by PMAIP1 or stabilized by the deubiquitinase USP9X [41]. We found that BIX-01294 up-regulated the expression of PMAIP1 and down-regulated the level of MCL1 and USP9X. In addition, in the cells transfected with *PMAIP1* siRNA, the levels of MCL1 and USP9X were augmented, while the cleaved caspases and PARP1 were decreased. These results suggest that BIX-01294 degrades the MCL1 through up-regulation of PMAIP1 and reduction of USP9X, which is consistent to our previous study that PMAIP1 upregulation reduces the availability of USP9X to MCL1, thereby promoting its ubiquitination and degradation, leading to the apoptosis of neoplastic cells [35]. Since DDIT3 also modulates other apoptotic proteins such as death receptors in the extrinsic pathway, we cannot exclude the role of extrinsic pathway in the apoptosis induction by BIX-01294 in bladder cancer cells. Therefore, further investigation is required to characterize as much as details involved in the BIX-01294-induced apoptosis.

In summary, our data demonstrate that BIX-01294 induces ER stress pathway, resulting in up-regulation of PMAIP1 and down-regulation of USP9X and MCL1, leading to apoptosis in bladder cancer cells. These findings may offer important insight into the molecular mechanism of BIX-01294, thereby accelerating its use in clinical scenarios which may help improve bladder cancer treatments.

## Abbreviations

EHMT2: Euchromatic histone-lysine N-methyltransferase 2
ER: Endoplasmic reticulum
UPR: Unfolded protein response
HSPA5: Heat shock 70kDa protein 5
ERN1: Endoplasmic reticulum to nucleus signaling 1
ATF4: Activating transcription factor 4
DDIT3: DNA-damage-inducible transcript 3
MCL1: Myeloid cell leukemia sequence 1
USP9X: Ubiquitin specific peptidase 9, X-linked
4-PBA: 4-phenylbutricacid
